# One crisis among many: Russia’s war in Ukraine and its implications for the MENA region

**DOI:** 10.1007/s42597-022-00081-9

**Published:** 2022-12-09

**Authors:** Clara-Auguste Süß, Irene Weipert-Fenner

**Affiliations:** 1 Research department “Intrastate Conflict” and research group “Radicalization”, Leibniz-Institut Hessische Stiftung Friedens- und Konfliktforschung (HSFK)/Peace Research Institute Frankfurt (PRIF), Baseler Str. 27–31, 60329 Frankfurt am Main, Germany; 2 Research department “Intrastate Conflict” and research initiative “ConTrust: Trust in Conflict—Political Life under Conditions of Uncertainty”, a joint research initiative between Goethe University Frankfurt and PRIF, Leibniz-Institut Hessische Stiftung Friedens- und Konfliktforschung (HSFK)/Peace Research Institute Frankfurt (PRIF), Baseler Str. 27–31, 60329 Frankfurt am Main, Germany

**Keywords:** Socioeconomic grievances, Food security, Violent conflict, Islamism, Mobilization, Sozioökonomische Missstände, Ernährungssicherheit, Gewaltsame Konflikte, Islamismus, Mobilisierung

## Abstract

Beyond the most immediate and devastating effects being felt by the local populace, the war in Ukraine is also having severe repercussions in other world regions, particularly the Middle East and North Africa (MENA), where we find significant effects on domestic and regional politics. This contribution discusses conflict potential along these two dimensions and how they are interrelated, including with global politics. First, the effects of the war were quickly felt at the domestic level via rising prices and shortages of (subsidized) staples. A deteriorating economic situation is heating existing societal conflicts that had led to waves of mass uprisings in the MENA over the last decade, which are very likely to re-appear in the near future. We discuss recent developments in light of existing knowledge about the nexus between socioeconomic grievances and (non-)violent mobilization. Second, we reflect on changes to regional and global politics and their impact on conflicts in the region. The EU’s sudden need for alternative oil and gas suppliers has improved the strategic position of Gulf countries, also vis-à-vis Iran in the rivalry over regional hegemony. This shift empowers such authoritarian regimes that support other autocracies as well as conflict parties in civil wars in the region. Instead of allying with either the West or Russia, the ruling elites’ relationship with global powers remains opportunistic. Cooperation with Russia and China might very well aggravate political grievances rather than remedy structural socioeconomic shortcomings—both of which will continue to fuel preexisting lines of conflict in the region.

## Abstract (de)

Der Krieg in der Ukraine hat zwar die unmittelbarsten und verheerendsten Auswirkungen für die Bevölkerung vor Ort, bringt aber auch schwerwiegende Folgen für andere Weltregionen mit sich. Dies gilt insbesondere auch für den Nahen und Mittleren Osten sowie Nordafrika („Middle East and North Africa“ [MENA]), wo sich der Krieg bereits erheblich auf die Innen- und Regionalpolitik auswirkt. Vor diesem Hintergrund diskutieren wir entsprechende Konfliktpotenziale und ihre Verflechtungen, auch mit der Weltpolitik. Die Auswirkungen des Krieges waren erstens in zahlreichen Ländern der Region schnell durch Preissteigerungen und Engpässe bei einigen (subventionierten) Grundnahrungsmitteln zu spüren. Die Verschlechterung der wirtschaftlichen Lage heizt bestehende gesellschaftliche Konflikte an, die im letzten Jahrzehnt zu Wellen von Massenaufständen in der MENA-Region geführt haben und die in naher Zukunft mit großer Wahrscheinlichkeit wieder aufflammen werden. Wir erörtern diese jüngsten Entwicklungen mit Blick auf bestehende Kenntnisse über den Zusammenhang zwischen sozioökonomischen Missständen und gewaltsamer sowie gewaltfreier Mobilisierung. Zweitens reflektieren wir Veränderungen in der regionalen und globalen Politik und ihre Auswirkungen auf Konflikte in der Region. Der plötzliche Bedarf der Europäischen Union an alternativen Öl- und Gaslieferanten verbesserte die strategische Position der Golfstaaten, auch im Wettkampf mit dem Iran um regionale Vorherrschaft. Gleichzeitig stärkt diese Verschiebung jene autoritären Regime, die andere Autokratien und Bürgerkriegsparteien in der Region unterstützen. Anstatt sich entweder mit dem Westen oder mit Russland zu verbünden, bleiben die Beziehungen der herrschenden Eliten zu globalen Mächten opportunistisch. Die Zusammenarbeit insbesondere mit Russland und China wird jedoch die politischen Missstände eher verschärfen, anstatt strukturelle sozioökonomische Defizite zu beheben, was bestehende Konflikte in der Region weiter anheizen könnte.

## Introduction

Shortly after Russia started its invasion of Ukraine, world prices for wheat, oil and gas skyrocketed. While Europe’s dependence on Russian energy exports was common knowledge, less known was how much numerous countries in the world rely on wheat imports from both Russia and Ukraine. Among the most vulnerable in this regard are Egypt, Sudan, Tunisia and Lebanon, which import between 50% and 90% of their annual wheat consumption from Russia and Ukraine (Arab Reform Initiative 2022). In Lebanon, this has led wheat prices to increase by almost 50% (World Food Program 2022a). High prices for energy have also affected a slew of critical imported food products in most countries in the Middle East and North Africa (MENA) (Lucente 2022). High energy and rising fertilizer prices are likewise affecting energy-intensive agricultural production, together contributing to runaway food prices. As of today, Yemen and Syria are already among the countries suffering from insufficient food supplies, with the same predicted for Palestine, Lebanon, Libya, Jordan, Morocco, Egypt, Tunisia and Algeria (in descending order of severity, World Food Program 2022b). As a result of this, a significant part of the MENA region is facing or will soon face food shortages and hunger.

In this contribution, we argue that the fallout from the Ukraine war is reinforcing preexisting conflict tendencies and we analyze the conflict potential at the domestic and regional levels. The first section aims to raise awareness of the man-made context in which these acute price increases and shortages occur (Sect. 2). We then consider the current crisis within ongoing and prospective (non-violent and violent) social mobilizations (Sects. 3 and 4, respectively) as well as in the light of the ongoing COVID-19 pandemic. In Sect. 5, we look at shifting regional conflict lines, consider how these are being affected by the recent changes in global politics and reflect on their impact on intrastate (non-)violent conflicts in MENA.

## The fallout of the Ukraine war: one crisis among many

The price hikes for basic goods and energy witnessed in the first months of Russia’s invasion of Ukraine were drastic, to say the least. Yet this has also happened in the context of years—if not decades—of recurrent inflation and price increases, particularly for food and basic goods. While inflation for food in Egypt, for instance, reached 22% in June 2022, a record high of 42% was previously witnessed in 2017 (Egypt Independent 2017). Since the outbreak of the COVID-19 pandemic, several countries in the region experienced average inflation rates of 20% for food (World Bank 2022a). Furthermore, inflation has been accompanied by stagnating wages, high unemployment rates, cuts to universal food and energy subsidies and the depreciation of many national currencies in the region (Loewe et al. 2021). After years of flat or negative economic growth rates since the 2007 financial crisis, the COVID-19 pandemic led to an economic downturn of up to 25% in per-capita GDP in 2020 in all but the resource-rich, high-income MENA countries (World Bank 2022b).

While exogenous shocks play a role in the current situation, explanations must take a long-term perspective on domestic and international political decision-making. Extremely high levels of food insecurity across MENA compared to other world regions are directly related to the effects of climate change and violent conflicts, such as in Iraq, Syria and Yemen (Belhaj and Soliman 2021). For other countries, such as Egypt, Jordan, Tunisia and Morocco, the reasons date back to political economy transformations and their shift from state-led to capitalist economies since the 1970s. These changes were induced by the international financial institutions (IFIs) and adapted to the logics of domestic politics by local elites. Such shifts brought about crony capitalist systems unable to create a sufficient number of decent jobs, particularly for people with higher education. Social spending on health, education and housing decreased, leading to an increase in informal solutions, particularly for the labor market (Cammett et al. 2015). Agricultural markets were oriented toward exports while cheap food imports reduced local food production (Zurayk and Gough 2014). These vulnerabilities became visible during the financial crisis of 2007/08, when prices and inflation increased sharply, leading to severe civic unrest in some MENA countries, most prominently in Egypt and Tunisia. In addition to recurrent droughts, 2010 saw record food prices on world markets, often used as an explanation for the outbreak of the Arab uprisings.

Since then, few structural changes have been undertaken to reduce these vulnerabilities—on the contrary. With the beginning of the Arab uprisings in 2011, the IFIs (particularly the International Monetary Fund [IMF]) returned to resource-poor countries of the MENA region. Among the standard repertoire of loan conditions, measures such as currency devaluation were included (Radwan 2020), which increased the import bill for basic goods on which many MENA countries rely and eventually led to worsening balance sheets for state budgets (for Tunisia for instance, see Observatoire Tunisien de L’Economie [OTE] 2019). Recent price hikes on the world markets have aggravated the matter, driving indebted countries to seek new IMF agreements. This comes at a time when universal subsidies for food and energy are being massively reduced and replaced by conditional cash-transfer programs. This shift in strategies, promoted by IFIs, has been criticized for unintentionally excluding up to 50% of the poor (Kidd et al. 2017) and deliberately excluding a middle class facing stagnating wages and rising inflation. As a result, and compared to pre-pandemic times, the World Bank forecasts that “7–8 million people will fall into extreme poverty” (Lopez-Acevedo et al. 2021, p. 21). While many governments in the region were cautious not to reduce subsidies for food and energy upon the outbreak of the Ukraine war, further reductions have been announced and will be part of new loan agreements.

In war zones across the region, recent price increases have added a huge financial burden on international aid organizations that provide basic goods for the most vulnerable in violent conflicts. The World Food Program (2022a), for instance, currently only has 24% of its required budget for Syria and 31% for Yemen. Again, the fallout from the Ukraine war is aggravating existing crises, among them the chronic underfunding of international humanitarian aid. This extreme increase in grievances and structural deficiencies is concurrently leading to heightened conflict potential. It is important to note that the objective existence of socioeconomic grievances (e.g. Collier and Hoeffler 2000) is not sufficient to explain mobilization, as they do not alone explain variation among different types of mobilization in some contexts or their widespread absence in others. The respective literature on social movements and radicalization suggests we also analyze movement/group characteristics and developments, their interaction with political and social actors, and, very importantly, the social meanings behind grievances (for an overview, see Weipert-Fenner 2021, p. 568)—which is the aim of the following two sections.

## Prospects of non-violent mobilization

Socioeconomic grievances have witnessed a steep increase in resource-poor MENA countries over the last years, more recently exacerbated by the global pandemic and now the war in Ukraine. The question at hand is whether these grievances will lead to and further fuel contention and conflicts across the region—be they violent or non-violent. In terms of non-violent contention such as contentious collective actions, we should acknowledge that, after the 2008 financial crisis and the ensuing economic crisis, the decade until 2021 was marked by a high number of protests around the globe. At the beginning of the COVID-19 pandemic, protests briefly declined by 35%, but they quickly returned in several countries and have been more intense than during the pre-pandemic period (ACLED 2021). This is in line with comparative research on post-pandemic protests that identifies a higher number of protests immediately following the outbreak of pandemics for a period of up to five years (Sedik and Xu 2020, p. 8). As COVID-19 caused the greatest recession since the Great Depression, we might expect even higher rates of unrest in the years to come.

The economic fallout from the Ukraine war has confronted already mobilized masses in every region of the world. This is particularly true for MENA countries: except for most of the resource-rich Gulf states, they have witnessed massive contention in the last decade (Weipert-Fenner and Wolff 2020). While public discourse in Europe tends to focus on the Arab uprisings in 2011, huge waves of protests have occurred from Morocco to Iran ever since. After the aforementioned COVID-19-related lull, there was a particularly large number of protests in Lebanon, Iraq and Tunisia (Garcia Chueca and Teodoro 2022, p. 4), while massive protests were staged in Sudan after the military coup in late 2021. Given the tense situation caused by high prices, many governments currently rely on concessions as well as price and export controls to prevent protests. Despite that, leading up to the summer of 2022, the first instances of unrest were already being witnessed in Iran and Iraq.

Protests cannot, however, only be explained by grievances alone but must consider a variety of additional influencing factors (Weipert-Fenner 2021). First, the framing of grievances is important, i.e. who is deemed responsible for shortages and price increases. In contrast to the first COVID-19 outbreak, the blame mainly seems to be attributed to national governments. In the MENA, as in many parts of the Global South, the Ukraine war is not seen as a global event but as a war in Europe (Hamzawy 2022), implying that socioeconomic grievances may continue to be perceived as an ongoing domestic policy failure. At the same time, while two-thirds of respondents were neutral in a recent YouGov poll with regards to the war, the survey indicated clear reservations about the West: the respondents held NATO (24%) and US President Biden (13%) responsible for the outbreak of the war, while only 16% pointed the finger at Russia—a finding the study interprets as “skepticism” and “deep […] mistrust of the West” (Arab News 2022). The question is whether authoritarian regimes will succeed in shifting the blame to the West in relation to the social crisis caused by the Ukraine war—as parts of the population in the Arab world seem to have bought into the Russian-sponsored discourse of an anti-imperialist struggle against Ukraine portrayed as a Western puppet (Salah 2022).

Second, when assessing conflict potential, the fact that mobilization has been widespread in the region means that skills and repertoires of contention as well as activist networks are already existent, all of which facilitate further protest. Finally, the specific nature of food riots, in particular, is very sensitive and hard to repress by brute force, especially as the so-called bread riots of the 1970s and 1980s are an important part of the collective memory. As bread has a very specific social meaning in the MENA, being a cornerstone of the daily diet among the general population as well as symbolic for life and making a living in Arabic language and culture (Martínez 2022), this context makes protests sparked by bread shortages or price increases relatively hard to repress. Moreover, they can serve as a starting point for broader demands, potentially leading to cross-class mass mobilization as seen during the Arab uprisings of 2011.

Earlier research on socioeconomic mobilization has shown that protests in this context have remained separate from Islamist groups—be they affiliated with the Muslim Brotherhood or Salafist groups (Weipert-Fenner and Wolff 2020, p. 23–24). As the conflict line between secular and Islamist forces is one of the most salient in the region, the conflict potential of socioeconomic grievances remains limited as long as the two conflict lines—socioeconomic and identity-based—do not overlap. This is, of course, a factor open to change and one that should be followed closely in the future.

## Radicalization, violent mobilization and intensification of violent conflicts

Research suggests that (violent) radicalization is more likely to occur when combined with (perceived) marginalization and grievances (for a synthesis of this point, see Poli and Arun 2019). Considering the worrisome economic developments and an exacerbation of preexisting socioeconomic maladies, the assumption that the war in Ukraine could also heighten the potential for radicalization across the MENA region does not seem far-fetched. To date, however, there has been no plausible empirical evidence to support this assumption. Furthermore, similar to non-violent mobilization, socioeconomic grievances do not necessarily lead to violent mobilization—as reflected in the COVID-19 pandemic. While the pandemic was, of course, a completely different context, its economic fallout also caused a sharp increase in socioeconomic grievances without leading to increased violent mobilization (see Süß 2021).

At the same time, radical actors, in general, are keen on adapting to new contexts and using these for their own purposes. This necessitates a close study of how Jihadists are interpreting the war and using it to frame their collective actions. As seen in cases such as the war in Syria, the so-called Islamic State in Iraq and Syria (ISIS) and the pandemic, various radical groups are prominently incorporating a new context into their (online) communication (ibid.). The war in Ukraine is no exception in this sense, as both al-Qaeda and ISIS were quick to comment on it. On the one hand, the Ukraine war has been portrayed as a war in Europe that Jihadists should profit from but not engage in directly. ISIS provided clear advice to its followers “to not get involved” in the war and to avoid taking sides in “Crusader-Crusader wars” (Shaker 2022). As such, a significant flow of foreign fighters to Ukraine or Russia from the MENA countries seems improbable.

On the other hand, ISIS encourages its supporters to take advantage of the high inflow of weapons and military equipment and appropriate them for their purposes. In line with similar recommendations by al-Qaeda, ISIS recommends that its supporters in the West utilize “Ukraine’s open borders and available arms to carry out attacks on ‘Crusaders’” (ibid.) in their homelands. While no evidence exists of such dynamics unfolding so far, the appropriation of weapons could pose the greatest immediate risk for potentially intensifying ongoing conflicts and insurgencies. The EU and NATO have already recognized this risk and established a “support hub” for better tracking and combating arms smuggling (Foy et al. 2022).

The way Jihadists frame the Ukraine war, as expressed in the online dimension of mobilization, connects to and builds on amplified grievances and the ability of radical actors to adapt. Some sources are speaking of a concurrent “information war” (Goldenziel 2022) taking place on traditional and social media: As Russia disseminates communication and, in particular, (dis)information campaigns via its high number of Arabic news outlets (Arab News 2022), it could contribute to hardening stances opposed to the West, potentially paving the way for vulnerabilities vis-à-vis Jihadist campaigns and recruitment efforts that also build on anti-Western narratives.

When considering violence, the impact of the war in Ukraine on current violent conflicts should certainly not be neglected either. Apart from the rapid deterioration of the humanitarian situation mentioned above, the Ukraine war appears to be a window of opportunity for actors to pursue their goals more assertively. The West’s and also Russia’s attention—hitherto heavily focused on Syria—is being directed elsewhere in part for the moment, to the benefit of terrorist groups such as ISIS, which is reportedly stepping up its attacks in Syria (Al-Kanj 2022). The same calculation seemed to be behind Turkey’s plans for a fourth operation there as well. Should Russia need to withdraw its military presence from the MENA—such as the engagement of Russian mercenary soldiers in Syria—due to a prolonged war in Ukraine, other actors might make take advantage of the ensuing power vacuum.

## Implications of the Ukraine war for regional politics

Common wisdom among MENA scholars dictates that regional and domestic politics are deeply intertwined. While complex regional politics in the MENA cannot comprehensively be discussed here, we would like to draw attention to the EU’s renewed heavy reliance on energy supplies from Sunni Gulf monarchies, and the implications of their empowerment at the regional level. Since 2011, the region was mainly structured along two conflict lines: 1) pro- and anti-Islamist forces and 2) Saudi-Arabia and its Sunni partners versus Iran and its allies. The first conflict line led to splits within the Sunni camp: while Saudi-Arabia and the United Arab Emirates (UAE) strongly opposed any Islamist rule in the region, Turkey and Qatar supported members or offspring of the Muslim Brotherhood in various political transformation processes (Fawcett 2019). It is fair to say that the anti-Islamist camp, with support for secular and often highly repressive republics, has maintained the upper hand. After years of conflict between Qatar and Saudi Arabia, reconciliation since January 2020 has proven stable, and Qatar has realigned itself with Saudi foreign policy (e.g. supporting the Al-Sisi regime in Egypt, Oommen 2022). Turkey has returned to its zero-problems foreign policy approach, seeking to be on good terms with Saudi Arabia and the UAE, as well as a neutral mediator between Russia and Ukraine (Ahmad 2022).

This realignment among Sunni states and the current high prices for oil and gas has provided Gulf monarchies a strong basis for continuing to support anti-Islamist authoritarian regimes in the region. Renewed economic and political weight, as expressed in long-term energy partnerships with the EU (European Commission 2022), is yet another path for pursuing a more autonomous and potentially assertive approach to regional politics. One major question is how an ever more powerful Saudi Arabia will act towards Iran in their conflict for regional hegemony. Given improved relations with Israel, another strong anti-Iran force in the region, the possible reelection of another “Trumpist” US president and a Russian president acting against the West’s interests in the MENA, one might worry whether any peaceful management of this regional rivalry can be found. This includes possibilities for a new nuclear agreement to be reached with Iran, which is currently at great risk.

We also need to closely observe how an even stronger Saudi-UAE alliance will affect the Yemen war and prospects for peace. Additionally, while the impacts on non-violent conflicts between Sunni and Shiite forces in the region (such as in Lebanon) are hard to foresee, the conflict potential is huge: Those mass uprisings in 2011 that turned violent all became highly internationalized civil wars due to support from regional powers. As the chances for further anti-regime mobilization are high (see Sect. 3), the empowerment of a pro-authoritarian, status-quo-oriented Saudi-UAE alliance would make their interference in domestic power struggles, and an eventual turn to protracted violence, more probable.

Saudi Arabia and the UAE are likely to continue supporting repressive autocracies and silencing the call for democracy in the region—using their regained strategic position vis-à-vis the EU to counter criticisms aimed at their foreign policy. At the same time, other global powers either lack the capacity or the will to restrain the Gulf monarchies in their regional politics. The Biden administration’s influence on the Saudi crown prince is rather limited (e.g. as reflected in the president’s failed attempts to persuade Saudi Arabia to increase oil production, Ebrahim and Al Lawati 2022). Russia, while being an ally of Iran and Syria, still exports arms to Saudi Arabia, the UAE and Egypt (Yahya 2022).

At the same time, MENA countries—in line with most countries in the Global South—do not have any intention of taking sides in the conflict between the West and Russia (Plagemann 2022). One example is their voting behavior regarding UN General Assembly Resolution ES-11/1 on Ukraine in March: While only six MENA countries did not vote in favor of the resolution that condemned Russia’s aggression, just one month later, the majority of the MENA countries abstained, voted against or did not participate in a vote on suspending Russia from the UN’s Human Rights Council (UN 2022). In general terms, MENA countries prefer to remain flexible in their alliances with global powers. This implies that cooperation with Russia is not ideological but rather strategic, as are relations with China. Beijing would benefit from the withdrawal of the U.S. in the region and is actively trying to expand its influence there—both economically and politically—without interfering by military means so far (Sun 2022). One common factor that remains true of all regional and global powers active in the MENA region is that none of them has offered any solutions to the multiple social and economic crises that exist—beyond short-term aid—but have rather contributed to aggravating political grievances.

## Conclusion

Against the background of the crises preceding the Ukraine war, caused by crony capitalism, climate change and COVID-19, this contribution has shown that the fallout from the Russian invasion into Ukraine is yet another (reinforcing) driver of conflict in the region. When considering the more direct and clear-cut impacts at the domestic level, we argue that, in the near future, the deteriorating economic situations and related grievances are more likely to lead to an upsurge of non-violent mobilization than to violent mobilization by Jihadist actors. For the regional level, we argue that the most important yet indirect impact so far is the further empowerment of Sunni Gulf monarchies around Saudi Arabia (as essential energy providers to Europe) and their anti-Islamist, anti-Shiite and anti-democratic agenda for the region. More resources and international legitimacy for the Saudi–UAE alliance can further reinforce domestic conflicts in resource-poor and conflict-ridden countries across the region. We conclude that scholars will need to link domestic and regional developments even more closely in future analyses—as should political decision-makers when evaluating cooperation such as new energy agreements. The effects of cooperation with a particular authoritarian regime may not only be problematic for the populace of that specific country but reinforce conflicts in other MENA countries as well.
